# Niosome as an Effective Nanoscale Solution for the Treatment of Microbial Infections

**DOI:** 10.1155/2023/9933283

**Published:** 2023-08-16

**Authors:** Mahmood Barani, Fatemeh Paknia, Maryam Roostaee, Batoul Kavyani, Davood Kalantar-Neyestanaki, Narges Ajalli, Alireza Amirbeigi

**Affiliations:** ^1^Medical Mycology and Bacteriology Research Center, Kerman University of Medical Sciences, Kerman, Iran; ^2^Department of Nanobiotechnology, Faculty of Biological Sciences, Tarbiat Modares University, Tehran 14115-154, Iran; ^3^Department of Chemistry, Shahid Bahonar University of Kerman, Kerman, Iran; ^4^Department of Medical Microbiology (Bacteriology & Virology), Afzalipour Faculty of Medicine, Kerman University of Medical Sciences, Kerman, Iran; ^5^Department of Chemical Engineering, Faculty of Engineering, University of Tehran, Tehran, Iran; ^6^Department of General Surgery, School of Medicine, Kerman University of Medical Sciences, Kerman, Iran

## Abstract

Numerous disorders go untreated owing to a lack of a suitable drug delivery technology or an appropriate therapeutic moiety, particularly when toxicities and side effects are a major concern. Treatment options for microbiological infections are not fulfilled owing to significant adverse effects or extended therapeutic options. Advanced therapy options, such as active targeting, may be preferable to traditional ways of treating infectious diseases. Niosomes can be defined as microscopic lamellar molecules formed by a mixture of cholesterol, nonionic surfactants (alkyl or dialkyl polyglycerol ethers), and sometimes charge-inducing agents. These molecules comprise both hydrophilic and hydrophobic moieties of varying solubilities. In this review, several pathogenic microbes such as *Staphylococcus aureus, Pseudomonas aeruginosa, Klebsiella pneumoniae, Plasmodium, Leishmania,* and *Candida* spp. have been evaluated. Also, the development of a proper niosomal formulation for the required application was discussed. This review also reviews that an optimal formulation is dependent on several aspects, including the choice of nonionic surfactant, fabrication process, and fabrication parameters. Finally, this review will give information on the effectiveness of niosomes in treating acute microbial infections, the mechanism of action of niosomes in combating microbial pathogens, and the advantages of using niosomes over other treatment modalities.

## 1. Introduction

One of the biggest challenges in medicine today is the treatment of deadly fungal, bacterial, and parasitic infections [1, 2]. The World Health Organization (WHO) reports that infectious diseases account for about one-third of all global deaths, with developing countries bearing the brunt of the burden. Malaria, tuberculosis, HIV/AIDS, and diarrhea are among the most prevalent infectious diseases that contribute to high mortality rates in these countries [3]. Infectious diseases can have a major economic impact on healthcare systems, especially in developing countries where resources are often limited. The treatment of infectious diseases can be particularly costly, especially for illnesses requiring prolonged treatment or medication. For instance, the expense of treating HIV/AIDS can be exorbitant for many patients, particularly those residing in low-income countries. Furthermore, these diseases can also contribute to lost productivity, as individuals are either unable to work due to illness or must care for sick family members. For example, the economic impact of malaria in sub-Saharan Africa has been estimated to account for as much as 1.3% of GDP [4]. The limitations of current treatments for infectious diseases, such as antibiotic resistance, toxicity, and low efficacy against certain pathogens, emphasize the pressing need to develop new treatment modalities and improve existing ones. Antibiotic overuse and misuse are significant contributors to the development of antibiotic-resistant bacteria, which poses a growing concern. Moreover, some drugs used in treating infectious diseases can cause severe toxic side effects, particularly when administered in high doses or over a prolonged period, which can adversely affect patients' quality of life. Similarly, certain treatments may not be effective against specific strains of a pathogen, leading to treatment failure [5]. To address these problems and challenges, in recent years, scientists have conducted extensively on nanoparticles as a valuable tool for treating microbial infections [6].

The application of nanotechnology in medicine, referred to as nanomedicine, is offering numerous exciting possibilities in healthcare [7–10]. Nanotechnology has shown great promise in the field of medicine, especially in drug delivery and targeted therapy. Nanoparticles can be designed to enhance the pharmacokinetics and pharmacodynamics of therapeutic agents, leading to improved efficacy and reduced toxicity [11, 12]. Moreover, nanoparticles can be functionalized with ligands or antibodies to precisely target specific cells or tissues, which can result in more accurate drug delivery and a reduced risk of off-target effects [13, 14].

Niosomes are nonionic surfactant-based vesicles, formed mostly by nonionic surfactant and cholesterol incorporation as an excipient that may facilitate drug absorption [15–17]. Recent advancements in niosome-based drug delivery systems have led to promising results in terms of improving drug efficacy, reducing toxicity, and enhancing targeted delivery [18, 19]. Niosomes are considered a substitute for liposomal phospholipids [20–23]. Niosomes can be divided into lipid-based and polymer-based carriers based on their main constituent [24, 25]. Many authors reported the use of niosomes as a valuable tool for the treatment of different disorders such as cancer and microbial infections [26]. For example, Anionic niosomes loaded with gallidermin were synthesized by Manosroi et al. They claimed that they are a superior topical antibacterial composition because gallidermin-loaded niosomes accumulate highly in the skin without the risk of having systemic effects. *Propionibacterium acnes* and *Staphylococcus aureus* were both resistant to the antibacterial effects of this formulation [27]. In another study, clarithromycin (CLR) niosomes incorporated in transdermal patches were developed by Alkilani et al. to address the problems with CLR stated above. The niosomal patch's flux (Jss) was more than 200 times greater than that of the traditional patch [28]. For example, studies conducted in 2016 by Sohrabi et al. showed that the niosomal formula increased antibacterial activity against *P. aeruginosa*. They were prepared from niosomes loaded with moxifloxacin as a potential carrier for topical antimicrobial delivery in chitosan gel [29]. Also, in a study conducted by Allam et al. in 2019, it was shown that the use of nanocompatible spherical niosomes increases antibacterial properties. In this study, vancomycin-loaded niosomes were used to treat ocular infections caused by methicillin-resistant *Staphylococcus aureus* (MRSA) infections. The results showed that this combination minimizes drug stimulation and improves patient treatment [30]. According to a 2021 study by Mansouri et al., using niosomes in combination with antibiotics will enhance their antibiofilm and antimicrobial properties. The potential of niosomes for biofilm penetration and controlled release was also evaluated [31]. In 2021, Eid et al. developed a niosomal azithromycin whose results indicate the effectiveness of nanocarriers in increasing the effect of azithromycin and improving the management of bacterial conjunctivitis [32]. In addition, studies conducted in 2022 by Mehrarya et al. have shown that niosomes have a good clinical effect in treating bacterial infections [33].

The purpose of this review is to discuss the application of niosomes as a nanoscale solution to address current challenges in the treatment of infectious diseases and antibiotic resistance. Ongoing research and development of niosomes as drug delivery systems for infectious diseases is crucial in addressing the persistent challenges posed by these illnesses. Advancements in niosome-based drug delivery have the potential to develop treatments that are more efficacious, have reduced toxicity, and are targeted for improved patient outcomes, ultimately decreasing the global burden of infectious diseases. By refining niosome-based delivery systems, researchers can create safer, more efficient treatments for antibiotic-resistant infections, emerging infectious diseases, and personalized medicine, thus enhancing the management and treatment of infectious diseases.

## 2. Global Concern about Microbial Infections

Today, drug resistance has become one of the significant challenges in the treatment and eradication of infectious agents. The phenomenon of drug resistance is observed in various groups of microorganisms such as fungi, bacteria, and parasites, increasing treatment costs, length of hospital stays, and mortality and morbidity. However, with the help of new methods such as nanodrugs, extensive studies have been used to identify effective drugs in recent decades. In the following of this section, we explain different types of resistant microorganisms to conventional drugs.

### 2.1. Bacterial Infections

#### 2.1.1. *Staphylococci*

*Staphylococci* are a broad genus of Gram-positive bacteria that colonize the skin and mucous membranes, such as most mammalian nasal cavities and the respiratory tract. Within the *Staphylococcus* genus, the most common skin commensal isolates are coagulase-negative staphylococci (CoNS), a large and heterogeneous family of staphylococcus, with the species *S. epidermidis*, *S. hominis*, *S. haemolyticus*, *S. capitis*, *S. lugdunensis*, and *S. warneri* [34]. CoNS are the reservoir for antimicrobial resistance genes and have an important role in nosocomial infections and promoting the development of methicillin-resistant *Staphylococcus aureus* (MRSA) [35]. Among CoNS, *S. epidermidis* is one of the major resident microflorae in the skin. Despite *S. epidermidis* being considered harmless but accompanied by other CoNS can be harmful [36]. *Staphylococcus aureus* is a causative agent of minor or self-limited skin infections including impetigo, folliculitis, carbuncles, furuncles, cellulitis, subcutaneous abscesses, and scalded skin syndrome (SSS) [37]. MRSA isolates are responsible for most cases of skin and soft tissue infection [38].

*Staphylococcus aureus* is a Gram-positive coccus that acts as a commensal microbiota in humans. It can also become an opportunistic pathogen and cause skin infection, respiratory infection, and food poisoning. *S. aureus* is also responsible for complicated urinary tract infections in hospitalized patients [39].

#### 2.1.2. *Pseudomonas aeruginosa*

*Pseudomonas aeruginosa* is an aerobic motile Gram-negative rod. It is a ubiquitous opportunistic pathogen that frequently causes disease, particularly in vulnerable hosts. *P. aeruginosa* is listed as a priority pathogen for the research and development of new antibiotics by the World Health Organization (WHO) which is the greatest threat to human health [40].

This bacterium is related to acute and chronic infections like lung infections, wound infections, and also as nosocomial infections. Infections are either created by local inoculation or following bloodstream infections. The most common infections originating from the skin by itself include green nail syndrome, toe web infection, hot tub folliculitis, hot hand-foot infection, and external otitis. *P. aeruginosa* skin infections are often related to water reservoirs [41]. Followed by bloodstream infections in immunocompromised patients, ecthyma gangrenosum and subcutaneous nodules are usually observed. In some skin infections like burn wound infections, necrotizing skin, and soft tissue infections occur in patients with burns and immunocompromised patients, *P. aeruginosa* can be observed [42].

#### 2.1.3. *Klebsiella pneumoniae*

*Klebsiella pneumoniae* is a member of the big *Enterobacteriaceae* family; it is a Gram-negative, nonmotile and facultative anaerobic bacilli. *K. pneumoniae* is currently regarded as one of the most important opportunistic pathogens causing hospital and community-acquired infections, particularly among immunocompromised patients and individuals hospitalized for a long time who use a lot of antimicrobial agents and be catheterized. Other infections related to this organism include pneumonia, liver abscess, meningitis, and bloodstream infections [43]. Many investigations show that antibiotic resistance among this organism has increased, which has become a great concern worldwide and makes a crucial problem in treating *Klebsiella* infections [44, 45].

#### 2.1.4. *Enterococcus*

*Enterococcus* spp. is belong to the group D Streptococcus system. It is a Gram-positive, facultatively anaerobic, and commensal coccus that lives in the gastrointestinal tract of humans. They can cause invasive infections if the balance of the microbiota is disrupted and cause different community- and hospital-acquired infections such as endocarditis, sepsis, urinary tract infections (UTIs), and meningitis [46]. The use of used-spectrum antibiotics and abdominal surgery are important risk factors for enterococcal infection [47, 48]. *E. faecium* and *E. faecalis* are the most frequently isolated species in the nosocomial environment. *E. faecium* is named a priority pathogen by the World Health Organization (WHO), a catalog of 12 families of bacteria that pose the greatest threat to human health [40].

#### 2.1.5. *Acinetobacter*

*Acinetobacter* spp. is a group of aerobic, Gram-negative coccobacillus in pairs with four different *Acinetobacteria*, including *A. baumannii*, *Acinetobacter pittii*, *Acinetobacter nosocomial*, and *Acinetobacter calcoaceticus* [49]. They are free-living saprophytic organisms widely distributed in different environments, including soil, water, vegetables, various parts of animals, and the human body, and also distributed in hospital environments [50]. *A. baumannii* has emerged as a major causing nosocomial infection, especially in intensive care units (ICUs) worldwide. It is a principal agent of ventilator-associated pneumonia; it can also cause the following infections: bloodstream, skin, and soft tissue, urinary tract infections (UTI), and meningitis (19). Risk factors for acquiring *A. baumannii* are long-term hospitalization in the ICU, immunocompromised individuals, use of medical devices like a catheter, smoking, alcoholism, diabetes mellitus, and COPD [50].

#### 2.1.6. *Mycobacterium Tuberculosis*

*Mycobacterium tuberculosis* (Mtb) is an acid-fast bacterial pathogen that causes tuberculosis (TB) and is responsible for 1.6 million human deaths annually. According to an estimated World Health Organization (WHO) 9.9 million people acquired ill with TB worldwide in 2020 [51].

TB is a highly airborne infectious disease that causes pulmonary TB at first and can affect other parts of the body to cause extrapulmonary TB afterward (21). The risk factors for acquiring infection are living in areas of the world with high rates of TB, infants and children under 5 years of age, and immunocompromised patients. Different levels of drug resistance in TB strains, such as multidrug resistance (MDR) and extensive drug resistance (XDR), are emerging [52]. Its complex and hydrophobic cell envelope prevents the influx of many drugs into the bacterial cytoplasm [53]. Drug resistance to TB makes it a significant challenge to therapy and control programs and makes it a major threat to global public health. Researchers estimated that the COVID-19 pandemic could increase up to 20% of deaths due to TB over 5 years [54].

### 2.2. Parasitic Infections

#### 2.2.1. *Plasmodium*

*Plasmodium* parasites are amoeboid intracellular parasites that cause malaria illness to distribute by female mosquitoes of the genus Anopheles. According to the latest World Malaria Report, 241 million malaria cases were detected, which estimated the number of malaria deaths at 627 000 in 2020 (24). Plasmodium has 5 species, but among them, 2 species, *P. falciparum* and *P. vivax*, are causes of malaria, and among them, *P. falciparum* malaria is prevalent [53, 55]. Malaria is a disease with chills and fever, anemia, and splenomegaly. This was observed in areas of the world with high rates of vector and immunocompromised patients [56].

#### 2.2.2. *Leishmania*

The protozoan parasite *Leishmania* can lead to leishmaniasis. Leishmaniasis is a vector-borne disease in which parasite species and phlebotomine sand fly coevolve to transmit the disease. Depending on the parasite species, leishmaniasis has a wide spectrum of manifestation: cutaneous leishmaniasis (CL), mucosal leishmaniasis (ML), disseminated or diffuse cutaneous leishmaniasis (DCL), and kala-azar or visceral leishmaniasis (VL) that caused by species such as *L. major*, *L. braziliensis*, or *L. guyanensis* [57, 58].

#### 2.2.3. *Schistosoma*

*Schistosoma*, commonly known as blood flukes, is responsible for a wide variety of diseases such as intestinal schistosomiasis, hepatosplenic schistosomiasis, and urogenital schistosomiasis, especially prevalent in tropical and subtropical regions. Among these parasites, *Schistosoma haematobium* (agent of UTIs) and *Schistosoma mansoni* are the most common pathogen species. Schistosomiasis or bilharzia is frequent in poor communities and is very debilitating. Schistosome infection occurs by contact with freshwater contaminated by cercariae in humans. Cercariae are an infectious stage of schistosomes that are released by the intermediate host snail [59].

#### 2.2.4. *Trichomonas vaginalis*

*Trichomonas vaginalis* is belong to a protozoan parasite that harbors flagella. This parasite is the causative agent of trichomoniasis, the most prevalent sexually transmitted infection in women aged 51-60 years worldwide [60].

#### 2.2.5. *Trypanosoma*

*Trypanosoma* is a genus of kinetoplastids which is a group of uniflagellated and obligate parasitic protozoans transmitted by a vector. Like other organisms in the order kinetoplastida, they are characterized by a modified mitochondrial genome known as the kinetoplast. The life cycle of this protozoan alternates between a mammal host and an insect vector, the tsetse fly. In an insect vector, they are found in the intestine, but in a mammalian host, they are in the bloodstream or an intracellular environment. Depending on Trypanosoma species, a spectrum of diseases such as fatal human diseases sleeping sickness (*T. bruci*) and Chagas disease (*T. cruzi*) can be observed. Human African trypanosomiasis (HAT) like *Trypanosoma brucei gambiense* (*T. b. gambiense*) and *Trypanosoma brucei rhodesiense* (*T. b. rhodesiense*) is very infecting. They can live in the bloodstream and reach the liver, spleen, and heart and can also able across from the blood-brain barrier and enter the central nervous system [61, 62].

### 2.3. Fungal Infections

#### 2.3.1. *Candida* spp.

*Candida albicans* and non-*C. albicans Candida* (NACA) species are normal microbial flora in the gastrointestinal tract and vagina of many healthy people. According to many investigations, some conditions, like immune deficiencies change the balance between *C. albicans*, NACA yeasts, and the other host normal flora. In this condition, the commensal *Candida* may convert into opportunistic pathogenic microorganisms and create a diverse group of infections called Candidiasis that involves the skin, nails, mucous membranes, gastrointestinal tract, and UTIs in the host [63]. The presence of Candida species in urine is called candiduria, which needs careful interpretation. Some risk factors of candiduria include female sex, urological and nonurological surgery, patients hospitalized in the intensive care unit admission, indwelling devices such as catheters, and recent use of broad-spectrum antibiotics [64]. The risk factors for candidiasis are diabetes mellitus, immunosuppressant therapies, antibiotic or steroid therapy, obesity, and severe diseases like HIV [64].

*Candida auris* is a new fungal species that have emerged in healthcare facilities. It is a multidrug-resistant pathogen that can cause nosocomial outbreaks of invasive fungal infections. This yeast can colonize the skin and other body sites asymptomatically and persist on surfaces and equipment [65, 66].

#### 2.3.2. *Aspergillus fumigatus*

*Aspergillus fumigatus* is a ubiquitous and adaptive fungus. It spreads by asexual sporulation and conidia production. Depending on the host's immune status, different manifestations of aspergillosis, ranging from allergic and chronic infections to acute invasive aspergillosis (IA), can be observed. Lung infections due to *A. fumigatus* is caused by the inhalation of airborne conidia present in the environment [67]. Aspergillus-related lung disease occurs when the respiratory tract's normal flora composition is disturbed or in patients with immunosuppressive therapies, so it is not a primary pathogen [67].

#### 2.3.3. *Cryptococcus neoformans*

*C. neoformans* are ubiquitous fungi that cause one of the crucial invasive opportunistic fungal pathogens. It is responsible for more than 220,000 infections and 180,000 deaths annually [68]. Wake human-like immunocompromised individuals, particularly those who have HIV, can be infected with inhalation of spores which results in lung infections. In these people, fungi cannot normally be clear for dissemination in the body and get access to the CNS hematogenously [69].

#### 2.3.4. Dermatophytes

Dermatophytes are filamentous and keratinous fungi that live in keratin-rich areas such as soil or human or animal tissues (skin, nails, and hair). Dermatophytes contain three genera: *Trichophyton* spp., *Microsporum* spp., and *Epidermophyton* spp. Another classification of dermatophytes is based on the source of infection: (i) anthropophilic transmitted by direct contact from one human to another, (ii) zoophilic is the fungi transmitted from animals, domestic or wild, to human, or other animals, and (iii) Geophilic, living on keratinous materials as saprophytes, can transmit to humans after contact with contaminated soil. The disease caused by dermatophytes is called dermatophytosis or tinea. Dermatophytosis like tinea pedis (athlete's foot), tinea unguium (onychomycosis, nail infections), tinea cruris (ringworm of the groin), tinea capitis (ringworm of the scalp), and tinea corporis (ringworm on the trunk) usually occurs in both genders at different ages [70]. The risk factors of dermatophytosis are sharing of fomites, uncontrolled access to infected animals, immunocompromised patients, and socioeconomic factors [71].  Table 1 shows a summary of microbial infections.

## 3. Niosome Nanocarriers

Niosomes are unilamellar/multilamellar vesicular structures based on nonionic surfactants that are formed by self-assembly processes in aqueous solutions [74, 75]. The structure of niosomes enables them to carry a variety of hydrophilic or hydrophobic compounds with high loading capacity and excellent encapsulation efficiency. In addition, targeted drug delivery is achieved through niosome surface engineering [76]. In the following section, we discuss the structure, properties, synthesis methods, and characterization techniques of niosome nanocarriers. Eventually, the applications of niosomes in the treatment of microbial infections are reviewed.

### 3.1. Structure and Properties

**Figure 1:**
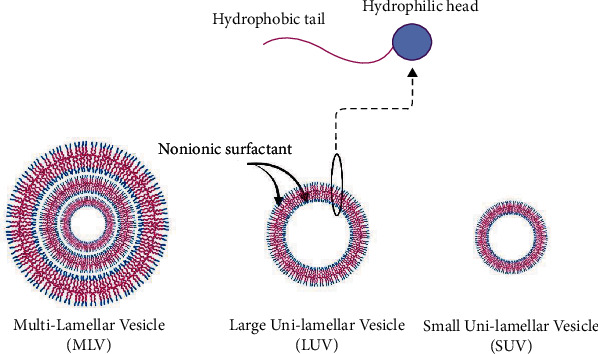
Typical classification of niosomes based on the bilayers number.

Mostly, nonionic surfactants and cholesterol or their derivatives are used to synthesize niosomes. Examples of surfactants used in the preparation of niosomes are listed in Table 2. By self-aggregation of monomeric units in an aqueous solution, concentric bilayer vesicles are formed. Hydrophobic chains are embedded within lipid bilayers, and the hydrophilic ends are exposed to the aquatic environment [22, 77, 78]. Accordingly, niosomes are classified into three groups based on the number of bilayers or their size: small unilamellar vesicles (SUVs, 10-100 nm), large unilamellar vesicles (LUVs, 100-3000 nm), and multilamellar vesicles (MLVs, >5 *μ*m) [18, 79, 80] (Figure 1). In addition, another type of classification of niosomes is based on their components.

#### 3.1.1. Aspasomes

Combining ascorbyl palmitate (ASP) with cholesterol and a negatively charged lipid leads to the formation of aspasome vesicles [81]. The antioxidant potential of ASP in the structure of these vesicles is an applicable and unique feature of aspasomes [82].

#### 3.1.2. Proniosome

Proniosomes are vesicular structures based on a dry powder formulation coated with a water-soluble nonionic surfactant. Hydration of these structures creates niosomes. Proniosomes are more stable than niosomes and exhibit higher bioavailability [83–85].

#### 3.1.3. Polyhedral Niosomes

These vesicles have spherical and nonuniform structures [86]. Polyhedral niosomes are mainly synthesized from a combination of Solulan C24 and hexadecyl diglycerol ether (C_16_G_2_) with or without cholesterol [87].

#### 3.1.4. Bola Niosomes

The amphiphilic structures of bola (*α*,*ω*-hexadecyl-bis-(1-aza-18-crown-6)) [18] are composed of a long alkyl chain linked to two azacrown ether units. These structures, in combination with cholesterol and span, form bola niosomes during the self-aggregation process [88]. Low critical micelle concentration and excellent surface tension are the advantages of bola surfactants [82].

#### 3.1.5. Elastic Niosomes

These vesicles are mainly synthesized from a combination of cholesterol, surfactants, water, and ethanol [82]. The most prominent feature of this group is their high flexibility, which enables them to pass through pores smaller than their diameter [89].

#### 3.1.6. Niosomes in Carbopol Gel

Niosomes prepared in situ in gel systems (carbopol-934/carbopol-940 gel) increase the availability of the drug loaded into the nanocarrier and improve its blood circulation time. In addition, these structures are associated with improved mucosal adhesion [77, 90].

### 3.2. Synthesize and Characterization

#### 3.2.1. Methods of Preparation

Different techniques for fabricating and preparing niosome nanocarriers have been introduced, and each method has its advantages. Notably, the fabrication method used is effective on the final properties of the niosome [99]. In this section, an overview of different methods for niosome preparation is provided.

*Thin film hydration (TFH) or handshaking method*: in this method, a mixture containing cholesterol and nonionic surfactant is dissolved in an organic solvent. Next, a thin film is formed using a rotary evaporator, while the solvent evaporates. At a temperature higher than the gel-liquid transfer temperature, the formed film is hydrated by adding a buffer, and the milky suspension of the niosome is obtained as the final product [100, 101].

*Ether injection (EI) method*: in this technique, cholesterol and surfactants are dissolved in a mixture of ethanol/diethyl ether and gently injected at 0.8 mL/min using a needle into a buffer that is preheated to 60°C or above the organic solvent boiling point [102, 103].

*Bubble method*: in this technique, a mixture of cholesterol, surfactant, and buffer is transferred to a three-necked glass flask without the need for organic solvents. Nitrogen and water-cooled reflux are available through the second and third necks, respectively. The temperature is controlled by a thermometer located in the first neck of the flask. Dispersion of niosome components is provided using a high-shear homogenizer. Then, nitrogen gas is slowly passed through the solution at 70°C [22, 104].

*Reverse phase evaporation (REV) method*: in the REV method, surfactants and cholesterol are dissolved in an organic solvent such as chloroform. A rotary evaporator is used to evaporate the solvent and form a lipid thin film. The resulting thin film is sonicated after adding a mixture containing ether and chloroform. Complete removal of organic solvents is achieved using a rotary evaporator and nitrogen gas [105–107].

*Microfluidization method*: this method is developed based on mixing two fluid streams in microchannels [80]. The solution is pumped at a rate of 100 mL/minute and circulated in a cooling loop to remove the heat generated in the microfluidization process. Recently, this method has received much attention due to its reproducibility and the production of small unilamellar vesicles [108].

*Proniosomal method*: cholesterol, surfactants, and ethanol are transferred to a vial and heated in a water bath. Then, while the above solution is in a hot water bath, an aqueous phase is added to produce a clear solution. In the following, the niosomes are formed by the hydration of the proniosomal gels. Niosome preparation is achieved by adding a buffer to the vials containing proniosomes and stirring with a homogenization shear in a water bath at 70°C [109, 110].

*Micelle solution and enzymatic method*: in the preparation of niosomes during an enzymatic process of mixed micellar solution, the ester bonds are cleaved by esterases, which are followed by the breakdown of products such as polyoxyethylene and cholesterol. Multilamellar niosomes are obtained by adding diacetyl phosphate and similar lipids to compounds resulting from the enzymatic process [111].

*Heating method*: in this method, cholesterol and surfactants are hydrated separately in an aqueous solution. Then, the cholesterol solution is heated under a nitrogen atmosphere for 30 min at 120°C. Surfactants are added to a cholesterol solution that is precooled to 60°C while stirred constantly. Finally, the prepared niosomes are stabilized at room temperature for 30 min under a nitrogen atmosphere [112, 113].

*Sonication method*: in this technique, a mixture containing cholesterol and nonionic surfactants is added to a buffer. Then, a suspension of the mentioned compounds is sonicated at 60°C for 3-4 min to produce niosomes [114].

Each of the techniques introduced in the preparation of niosomes has advantages and disadvantages over the others. For example, some studies have shown that the entrapment efficiency (EE) of niosomes prepared by the EI method is higher than the niosomes synthesized by the TFH technique. Moreover, the use of the microfluidization technique leads to the synthesis of niosomes with more uniformity and smaller size than other methods [115]. Evidence suggests that the addition of a sonication step helps to form smaller and more homogeneous vesicles. Therefore, it is better to use a size reduction step in the preparation of niosomes following the initial hydration process [114, 116].

#### 3.2.2. Characterization Techniques

To develop the use of niosomes for *in vitro* and *in vivo* studies, it is necessary to evaluate their quality and characteristics. Some of these parameters including size, morphology, surface charge are summarized in Table 3. The three parameters of niosome purification, EE, and *in vitro* release are discussed in more detail below.

*Niosome purification*: the vesicle purification process is essential for the removal of impurities and unencapsulated drugs. Hence, purification methods such as dialysis, gel filtration chromatography, and centrifugation can be mentioned [117]. The dialysis method uses dialysis bags, which are a kind of semipermeable membrane and are based on the diffusion phenomenon. Niosomes are dialyzed against water or a saline solution [118, 119]. In addition, the process of purification of niosomes by gel filtration chromatography method is performed using Sephadex G25, G50, and G75 and washing buffers such as HEPES [120]. One of the easiest ways to purify niosomes is to use a centrifuge and ultracentrifuge (the drug-loaded niosomes precipitate, and the supernatant contains the free drug). Filtration with membrane filters is also common in the purification of niosomes [82, 121, 122].

*Determination of EE*: this parameter determines the percentage of drug molecules entrapped in the vesicle. The most common method for determining the EE% is the dialysis bag method. In this process, the drug-loaded niosomes are transferred to a dialysis bag and placed in a buffer solution. After 24 h, a defined amount of buffer solution containing the unentrapped drug is collected. Then, the amount of nonloaded drugs is quantified using HPLC and UV-visible spectroscopy at a given wavelength. In addition to the above method, with the use of filtration and centrifugation, the free drug and the drug entrapped in the vesicle can be separated. The EE% is calculated by the following equation, based on the quantitative difference between the total added drug and the unentrapped drug [18, 123, 124].

(1) $$\begin{array}{c} \%\text{Entrapment efficiency }\left(\text{EE}\right)=\frac{\text{Total drug }\left(\text{Wt}\right)-\text{Unentrapped drug }\left(\text{Wt}\right)}{\text{Total drug }\left(\text{Wt}\right)}\times 100. \end{array}$$

*In vitro release investigations*: the study of *in vitro* drug release helps to identify the systemic circulatory characteristics of the drug and determine the optimal prescribed dose at various time intervals. *In vitro* drug release is mainly studied using a dialysis bag. The drug-loaded niosomal nanocarriers are immersed in a phosphate buffered saline (PBS) at the appropriate pH after transfer to a dialysis bag. This system is placed in the shaker or under magnetic stirring (37°C, 100 rpm). The buffer solution is removed after specified times and replaced with the same amount of fresh PBS. The collected samples are then analyzed to determine the released drug concentration [125, 126].

### 3.3. Application of Niosomes for the Treatment of Microbial Infections

Considering the prevalence of microbial infections and increasing antibiotic resistance, serious measures to maintain the health of human communities seem necessary. Nanocarriers have been introduced as one of the most appropriate approaches to increase the stability and controlled delivery of drugs and antimicrobial agents [137]. Among the types of nanocarriers, niosomes with their unique structure and the ability to encapsulate hydrophilic and hydrophobic drugs simultaneously are considered efficient toolsets in drug delivery [138]. Easy synthesis, available and cheap raw materials, biocompatibility, and excellent solubility are other advantages of niosomes [139]. Niosomes improve the efficacy of antibiotics and other anti-infective agents through several mechanisms. Firstly, their small size (typically less than 500 nm) allows them to penetrate biological barriers, such as cell membranes, and accumulate at the site of infection [140]. Additionally, niosomes can be functionalized with specific ligands or antibodies, targeting the pathogen and improving drug accumulation while reducing off-target effects. The lipid bilayer structure of niosomes is similar to cell membranes, facilitating the incorporation of drugs into the niosome membrane [141]. Moreover, niosomes' hydrophobic nature enables them to fuse with bacterial cell membranes, allowing for efficient drug delivery into these cells. Finally, the use of cationic niosomes can enhance drug accumulation by electrostatically interacting with negatively charged bacterial cell walls [142]. To achieve effective delivery of drugs to bacteria using niosomes, it is essential to carefully choose appropriate ligands or make modifications to the surface of the nanoparticles. This is because the ability of the nanoparticles to enter the bacterial cells and deliver the drug depends on their binding and uptake by the cells. Specific ligands or antibodies can be added to the surface of the niosomes to enhance their binding and uptake by the bacteria, thereby increasing drug delivery to the targeted cells. Examples of effective ligands include chitosan, peptides like lactoferrin and colicin, antibodies, and antibody-derived peptides, as well as certain plant extracts like curcumin and berberine. The selection of the appropriate ligand depends on the specific bacteria being targeted and the desired therapeutic outcome [143, 144]. This section discusses the role of niosomes in the management and treatment of microbial infections.

#### 3.3.1. Niosomes and Bacterial Infections

Management of bacterial infections requires the administration of large amounts of antibiotics; the widespread and systemic distribution of these drugs is associated with several side effects [33]. Studies have demonstrated the efficacy of niosomes for the targeted delivery of antibiotics to infection-specific sites. In this way, many authors reported the enhanced potential of niosomes in treatment of infection disease. For instance, a research team designed a niosomal nanocarrier loaded with ciprofloxacin. The study demonstrated a significant reduction in biofilm formation by methicillin-resistant Staphylococcus aureus (MRSA) when treated with a niosome-loaded ciprofloxacin nanocarrier [145]. In another study, a niosome delivery system was used to increase the cellular uptake of a locked nucleic acid-2′-O-methyl hybrid antisense oligonucleotide (LNA-2′-O-Me hybrid-ASO) that targets the acpP (acyl carrier protein P) gene in *Pseudomonas aeruginosa* isolates. The researchers in this study believe that because the acpP gene is critical for bacterial cell wall construction, delivery of anti-acpP against *P. aeruginosa* using niosomes is appropriate and efficient for antisense approaches and antibiotic alternatives [146].

Drug-resistant (DR) *Klebsiella pneumoniae* is recognized as a serious threat in hospital settings. The eligible effect of niosome-encapsulated azithromycin in comparison with soluble azithromycin in the inhibition of DR *K. pneumoniae* has been reported [147]. In another interesting study, a nanocomposite consisting of niosomes, zinc oxide nanoparticles, and collagen was synthesized, and its antibacterial effect was investigated. The findings of this study expressed the high potential of nanocomposites in inhibiting Gram-positive and Gram-negative pathogens due to the antimicrobial activity of zinc oxide nanoparticles and the integration of niosome in bacterial cell membranes [148]. According to the researcher's opinion, the effectiveness of antibiotics is not significant due to the control mechanisms and limited permeability of the bacteria's outer membrane, whereas, the interaction of niosomes with bacteria through processes such as bacterial membrane integration, contact diffusion, and adsorption is associated with increased efficacy of antimicrobial agents and accumulation of drugs at the site of infection [149].

**Figure 2:**
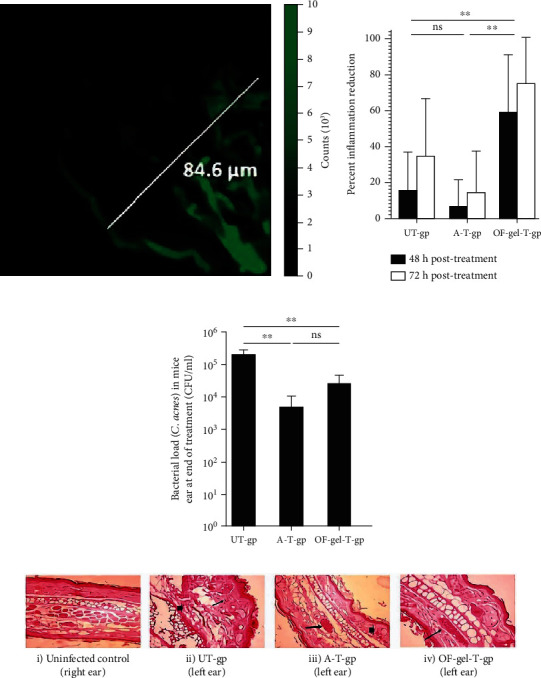
(a) The mouse skin fluorescence photomicrograph was taken by confocal laser scanning microscopy. (b) Percent inflammation reduction in UT-gp, A-T-gp, and OF-gel-T-gp after 48 h and 72 h. (c) *Cutibacterium acnes* counts in mouse ears for UT-gp, A-T-gp, and OF-gel-T-gp. (d) Digital photographs for histopathological assessment of mouse ears: (i) normal histology of control sample without edema and tissue inflammation, (ii) untreated infected ear with severe dermal necrosis (arrowhead), malpighian layer atrophy, and lymphocytic infiltrate (arrow), (iii) infectious ear treated with Aknemycin® with dermal hemorrhage (arrowhead) and congested blood vessel (arrow), and (iv) normal histology of OF-gel-treated infected ear with basal layer and congested dermal blood vessel (arrow), reprinted with permission from ref. [150]. Abbreviations: UT-gp: untreated group; A-T-gp: Aknemycin®-treated group; OF-gel-T-gp: optimized formula-gel-treated group.

A research group designed the dapsone niosomal nanocarrier, and after preparing its optimal formula gel, the *in vivo* activity of this niosomal system against *Cutibacterium acnes* was evaluated. The results of their studies confirmed the designed system penetration in different skin layers. In addition, the mouse model treated with the optimized formula gel exhibited increased recovery as well as a considerable reduction in inflammation compared to other treatment groups. Hereupon, the introduced nanocarrier can be promising for topical acne treatment (Figure 2) [150].

A recent study investigated the potential of niosome-encapsulated imipenem for the treatment of antibiotic-resistant bacterial infections. The researchers prepared various formulations of the drug and tested their effectiveness against *Staphylococcus epidermidis* isolates that were resistant to methicillin and capable of forming biofilms. The F1 formulation (Span 60 + Tween 60 + cholesterol) of niosomal imipenem was found to prevent biofilm growth and reduce the expression of certain biofilm genes while also reducing the minimum inhibitory concentration and minimum biofilm inhibitory concentration by 4-6 times. Importantly, the F1 formulation showed no toxicity to human cells at all concentrations tested. These findings suggest that niosome-encapsulated imipenem could be a promising new strategy for treating antibiotic-resistant bacterial infections [93].

Also, niosomes composed of two surfactants (Tween 85 and Span 80) without cholesterol were found to entrap ciprofloxacin, increase its stability, and induce inhibition of biofilm formation on *Escherichia coli* and *Staphylococcus aureus* [151].

The above-mentioned studies demonstrate the potential of niosome-based drug delivery systems for targeted antibiotic and anti-inflammatory treatments. The researchers in these studies suggest that the interaction of niosomes with bacteria through processes such as membrane integration, contact diffusion, and adsorption is associated with increased efficacy of antimicrobial agents and accumulation of drugs at the site of infection.

#### 3.3.2. Management of Fungal Infections

Considering the long duration of common treatments for fungal infections, the subsequent side effects and systemic toxicity are unavoidable [18]. Advantages from the features of drug delivery nanosystems are associated with reducing typical therapies' side effects and improving drug efficacy. Recently, niosomal nanocarriers have been considered for their biodegradability, low toxicity, resistance to oxidative degradation, and enhanced half-life of entrapped antimicrobial agents [152, 153]. For instance, Barot et al. designed and formulated a gel-based niosome loaded with farnesol to treat oral candidiasis caused by *Candida albicans*. The results revealed the biocompatibility, increased drug penetration, and antifungal activity of the proposed formulation [154].

In another study, a dual-purpose drug delivery system was designed to treat ocular keratitis infection. The effectiveness of natamycin-loaded niosomes in the ketorolac-tromethamine gel system against *Candida keratitis* was evaluated on rabbit models. The results of the research showed high sensitivity of *C. keratitis* to the optimized formula, desirable bioavailability, and improved corneal drug permeability. In addition, due to the anti-inflammatory activity of ketorolac tromethamine, the inflammation caused by the infection was also reduced [153]. Conventional treatments using natamycin, as an ophthalmic antifungal drug, are associated with high side effects and low success due to the requirement for high-dose and long-term treatments. Hence, the development of safe and effective treatment methods is seriously needed [153, 155].

The formation of biofilms is closely related to nosocomial infections. Microorganisms based on biofilms act as a potential source of infection because of their antibiotic resistance properties. *C. albicans* is known as an opportunistic nosocomial pathogen, and typical treatments have failed to suppress infection caused by this fungal pathogen. Using drug carriers with controlled and targeted delivery of antimicrobial agents moderates side effects and existing challenges [156–158]. As an example, a research group examined the antibiofilm effect of amphotericin B-loaded sophorolipid-based niosomal nanocarrier against *C. albicans*. According to the reported results, more viable cells were observed in the biofilm treated with amphotericin B compared to the untreated sample, while biofilm treated with niosomal formulations exhibited a significant reduction in complexity. Therefore, this study demonstrates the efficacy of niosomal nanocarrier in the management of *C. albicans* fungal infections [158].

In another interesting research, a niosome carrier was synthesized to enhance the antimicrobial activity of propolis (a resinous substance prepared by honeybees from herbal sources with antimicrobial, antioxidant properties, etc.). Niosome-based drug delivery systems enhance the antimicrobial effects of propolis by increasing its solubility, permeability, diffusion, and durability in the skin layers. In this study, after determining the propolis polyphenolic content, preparation of niosomes by the ethanol injection method, and characterization of drug-loaded niosomes, the antifungal and antibacterial potency of the designed formulation was investigated. According to the reported results, the minimum inhibitory concentration (MIC) of propolis-loaded niosomes against *S. aureus* and *C. albicans* was lower than propolis extract. In the following, to increase skin durability and facilitate topical application, carbopol-P934 gel was used to formulate the niosome systems [159].

Overall, these studies highlight the potential of niosomes as a promising drug delivery platform for various fungal infections, offering increased efficacy and reduced side effects [160].

#### 3.3.3. Parasite Infection Treatment with Niosome

In addition to extensive experimental studies on the use of niosomes for the treatment and management of bacterial/fungal infections, valuable evidence has been presented to enhance the effectiveness of antiparasitic therapies using niosome vesicles. For instance, Elmehy et al. evaluated the effectiveness of ivermectin niosomal form against *Trichinella spiralis* infection. Active encysted larvae in raw meat eaten by human initiate the process of *T. spiralis* infection in the stomach and small intestine. The later steps of the infection are associated with striated muscle damage, severe inflammation in the brain, heart, and lungs, and eventually death. The therapeutic potential of oral drugs such as ivermectin in infection early steps have been confirmed; nevertheless, the low bioavailability of these drugs has seriously challenged their efficacy in the more advanced phases of infection [161, 162]. Consequently, in the present study, a niosomal drug delivery system was proposed to improve the therapeutic effects of ivermectin. They began their biochemical and histopathological studies after synthesizing niosome nanocarriers and ivermectin nanocrystalline. In summary, the results revealed that the reduction of inflammatory responses in the intestinal cells of mouse models treated with ivermectin niosomal form was more obvious than with ivermectin nanocrystalline. In addition, a significant reduction in encysted larvae and their capsule destruction was reported in the diaphragm muscle of mice exposed to ivermectin-loaded niosomes. The superiority of the ivermectin niosomal form is related to the high potential of niosomes in increasing bioavailability and maintaining sustained, and controlled drug release with the optimal dose [161].

As another valuable example, recently, niosome-based nanocarriers were used to increase the solubility and bioavailability of praziquantel in the treatment of schistosomiasis. The results of *in vitro* studies for the *Schistosoma mansoni* infection treatment demonstrated that praziquantel solution, drug-free niosomes, and praziquantel-loaded niosomes killed 10%, 30%, and 50% of adult parasites, respectively. In addition, the superior efficacy of the praziquantel niosomal form over the free praziquantel solution was also reflected in histopathological and immunohistochemical evaluations [163]. This eligible function of niosomes is related to the direct delivery of the drug through their integration or adsorption to the microorganism's surface [163, 164].

Several experimental studies have reflected the strong antiparasitic activity of drug-loaded niosomes against leishmaniasis [165–167]. For instance, a research team investigated the antileishmaniasis effects of glucantime, amphotericin B, and their combination in encapsulated niosomic form and free form against *Leishmania tropica*. In brief, according to the findings of this study, considering the IC_50_ values, the inhibitory activities of encapsulated drugs in niosomes were more pronounced than in nonniosomal forms. Furthermore, an increase in the population of apoptotic cells was reported in *L. tropica* treated with niosomal formulations. It has been shown that glucantime and amphotericin B encapsulated in niosomes have an enhanced effect in suppressing intracellular and extracellular forms of *L. tropica*. The researchers in this study believe that the proposed niosomal formulation could be promising in leishmaniasis therapy [167].

Taken together, the studies reviewed above suggest that niosomes hold great potential as a drug delivery system to enhance the therapeutic efficacy of drugs against parasitic infections. The benefits of using niosomes include improved bioavailability, controlled and sustained drug release, and targeted drug delivery, which can overcome the limitations of traditional drug delivery systems. Continued research and development of niosomal drug delivery systems could lead to the development of safer and more effective treatments for parasitic infections.

## 4. Challenges and Future Prospective

Despite the vast potential of targeted nanoniosomes, the conversion of this technology into an industrial form and its clinical development are challenging in several ways. For example, identifying targeting ligands is highly specialized, selective, and problematic. Developing and adapting simple, efficient, and renewable processes are also challenging for high-end industrial production. In addition, rapid optimization of the biophysicochemical properties of novel nanoparticles for maximum efficiency and loading efficiency, drug release kinetics, and stability of complexes is important and challenging. Thus, many efforts have focused on nanoparticle development through self-assembly, the use of high-efficiency processes to facilitate screening, and the optimization of large-scale industrial processes. An innovative strategy is needed to increase the engineering accuracy of targeted nanoparticles in a simple and scalable manner.

The potential effects of nanoniosomes on microbial infectious diseases have already been reported in many treatments for microbial infections. Nanoparticles with unique physicochemical properties make it possible to diagnose microbial disease with selectivity, high sensitivity, and fast readability. The release of antibiotics by niosomes reduces side effects. In addition, coating medical devices with antimicrobial nanomaterials effectively reduces the bacterial infection of the devices and the formation of biofilms on the surfaces of medical devices. However, despite these interesting achievements, the potential of nanotechnology in the management of microbial infections, especially in the field of antimicrobial therapy and vaccines, has not yet been realized [82, 83, 119, 168, 169].

Additional factors that need to be taken into account include the possibility that the medicine that is encapsulated in niosomes may seize up, build up, fuse, or leak during storage because of the dispersion form of niosomes. Comparing niosome formulations to liposomes, many studies generally found that niosome formulations had higher stability. According to scientific literature, storage stability can last up to 6 months for niosomes [170]. By the way, several authors recommended using a polymeric substance in the niosome's composition to improve stability and reduce leakage. For instance, the grafting of polymers into the lipid bilayer changes niosome permeability and physicochemical features such as drug loading and leakage while also increasing the hydrophilicity of the niosome and its subsequent durability in an aquatic environment. Additionally, it has been demonstrated that adding PEG to the niosome composition prevents the entrapped molecules from leaking out of niosome particles stored in a phosphate buffer solution (PBS) [171]. It is hoped that, in the future, these nanosystems, due to their unique structure and characteristics, will have wide applications with higher efficiency in the fields of nanomedicine and treatment. With the increasing development of antimicrobial nanomedicine, it can be expected that more products based on nanotechnology will be produced and can be used clinically in the future to manage various aspects of microbial infections.

## 5. Conclusions

In recent years, vesicular drug delivery systems have attracted a great deal of attention. Among these, niosomes have received special attention. In this review article, we first discussed the infections and challenges caused by fungi, bacteria, and parasites, then the use of niosomes in nanocomparison for the treatment of infections. In short, niosomes are a very effective tool for drug delivery in the treatment of infectious diseases and have a higher capability than conventional drug therapies. Niosomes are a suitable, targeted, and effective drug delivery system with the ability to load both hydrophilic and hydrophobic drugs. Surfactants as building blocks of niosomes play an important role in the formation and properties of these nanocarriers, so any advancement in the synthesis of new surfactants that are nontoxic, low-cost, biocompatible, and biodegradable will increase the efficiency of niosomes. Analysis of the results presented in this review points to the need for more research regarding the design and experimentation of niosomes with antimicrobial activities. Although some studies have shown progress in what parameters are important in the niosome synthesis, more investigation is needed to acquire more knowledge or set standard experimentation to converge in an appropriate methodology.

## Figures and Tables

**Table 1:** Summary of microbial infections.

Microorganisms	Type of infection	Risk factor	Ref.
Bacterial agents			
*Staphylococcus aureus*	(i) Skin infection (ii) Endocarditis (iii) Respiratory infection (pneumonia) (iv) Food poisoning (v) Complicated urinary tract infections (UTI)	(i) Immunocompromised patients (ii) Individuals hospitalized for a long time (iii) Indwelling devices	[39]
*Pseudomonas aeruginosa*	(i) Skin infection (ii) Respiratory infection (pneumonia) (iii) Urinary tract infections (UTI)	(i) Immunocompromised patients (ii) Cystic fibrosis patients (iii) Individuals hospitalized for a long time (iv) Indwelling devices	[42]
*Klebsiella pneumoniae*	(i) Pneumonia (ii) Liver abscess (iii) Meningitis (iv) Bloodstream infections (sepsis)	(i) Immunocompromised patients (ii) Individuals hospitalized for a long time (iii) Indwelling devices	[43]
*Enterococcus faecium*	(i) Endocarditis (ii) Bloodstream infections (sepsis) (iii) Urinary tract infections (UTI)	(i) Use of broad-spectrum antibiotics (ii) Abdominal surgery	[46]
*Acinetobacter baumannii*	(i) Bloodstream infections (sepsis) (ii) Urinary tract infections (UTI) (iii) Skin and soft tissue infection (meningitis)	(i) Individuals hospitalized for a long time in intensive care units (ICUs) (ii) Immunocompromised patients (iii) Indwelling devices	[50]
*Mycobacterium tuberculosis*	(i) Pulmonary TB (ii) Extrapulmonary TB	(i) Areas of the world with high rates of TB (ii) Infants and children under 5 years of age (iii) Immunocompromised patients	[52]
Parasitic agents			
*Plasmodium falciparum*	(i) Malaria (chills and fever, anemia, splenomegaly, acute kidney injury)	(i) Areas of the world with high rates of vector (ii) Immunocompromised patients	[56]
*Leishmania major*	(i) Cutaneous leishmaniasis (CL) (ii) Mucosal leishmaniasis (ML) (iii) Disseminated or diffuse cutaneous leishmaniasis (DCL) (iv) Kala-azar or visceral leishmaniasis (VL)	(i) Areas of the world with high rates of vector	[57]
*Schistosoma haematobium*	(i) Intestinal schistosomiasis (ii) Hepatosplenic schistosomiasis (iii) Urogenital schistosomiasis	(i) Poor communities	[59]
*Trichomonas vaginalis*	Sexually transmitted infection	(i) Women aged 51-60 years	[60]
*Trypanosoma bruci*	(i) Bloodstream infection (ii) Liver infection (iii) Spleen infection	(i) Rice culture (ii) Attendance at pirogue jettie	[72]
Fungal agents			
*Candida albicans*	(i) Candidiasis involves (ii) Skin and nail infection (iii) Mucous membrane infection (iv) Gastrointestinal tract infection (v) Candiduria	(i) Female sex (ii) Urological and nonurological surgery (iii) Patients hospitalized in the intensive care unit (iv) Indwelling devices (v) Immunocompromised patients (vi) Obesity	[73]
*Aspergillus fumigatus*	(i) Allergic (ii) Chronic and acute invasive aspergillosis	(i) Immunocompromised patients	[67]
*Cryptococcus neoformans*	(i) Respiratory infection (pneumonia) (ii) Meningoencephalitis	(i) Immunocompromised patients	[69]
*Dermatophytes*	(i) Dermatophytosis (skin and nail infection)	(i) Sharing of fomites (ii) Uncontrolled access to infected animals (iii) Immunocompromised patients (iv) Socioeconomic factors	[70]

**Table 2:** Examples of surfactants used in niosome nanocarriers.

Class of nonionic surfactant	Name of nonionic surfactant	Combined agent	Application of synthesized niosome	Ref.
Sorbitan fatty acid esters	Span 80	Clarithromycin	As a niosomal carrier with sustained and controlled release to increase drug bioavailability	[91]
	Span 60	Melittin	As a niosomal system to inhibit the bacterial skin infection	[92]
	Span 40	Imipenem	As a functional nanocarrier to prevent biofilm formation and reduce the antibiotic resistance of methicillin-resistant *S. epidermidis*	[93]
	Span 20	Lomefloxacin	As a niosome carrier to increase the bioavailability and antibacterial effects of lomefloxacin against ocular infections	[94]
Polyoxyethylene sorbitan fatty acid esters	Tween 80	Moxifloxacin	As an efficient nanocarrier for controlled delivery of antimicrobial agents against *P. aeruginosa*	[29]
	Tween 60	Azithromycin	As a niosomal system to improve bacterial conjunctivitis infection and targeted delivery of azithromycin	[32]
	Tween 40	Vancomycin	As a niosomal formulation to inhibit the biofilm formation and staphylococcal colonization on abiotic/nonbiological surfaces	[95]
	Tween 20	Hydrophilic silver nanoparticles (AgNPs)	As a niosome system with stable properties for efficient delivery of AgNPs, drugs, and biomolecules	[96]
Alkyl ethers	Brij 72, 78, & 92	Carvedilol	As a carrier in the form of proniosomal gel to increase the skin penetration of carvedilol	[97]
	Brij 52	Brimonidine tartrate	As an ocular drug delivery system in the form of a proniosomal gel to promote bioavailability and sustained release of brimonidine tartrate	[98]

**Table 3:** An overview of characterization techniques for niosomes.

Characteristic	Applied instrumentation	Additional notes	Ref.
Particle size	DLS, SEM, FE-SEM, TEM	—	[127, 128]
Zeta potential	DLS, Zetasizer	Zeta potentials higher than +30 mV and less than -30 mV are acceptable as a stable state of the niosomes	[82, 116, 127]
Morphology	SEM, FE-SEM, TEM, FF-TEM, cryo-TEM, NS-TEM, AFM, STM	—	[129–133]
Number of bilayers	AFM, SEM, TEM, NMR, SAXS, EDX, fluorescence polarization	—	[96, 130]
Vesicle stability	Microscopic techniques, DLS	Determining the EE % of niosomes at regular intervals helps to assess their stability	[134]
Structure and chemical bonding patterns	FTIR	—	[135, 136]

Abbreviations: DLS, dynamic light scattering; SEM, scanning electron microscopy; FE-SEM, field emission scanning electron microscopy; TEM, transmission electron microscopy; FF-TEM, freeze-fracture replication-electron microscopy; cryo-TEM, cryo transmission electron microscopy; NS-TEM, negative staining transmission electron microscopy; AFM, atomic force microscopy; STM, scanning tunneling microscopy; NMR, nuclear magnetic resonance spectroscopy; SAXS, small angle X-ray scattering; EDX, energy dispersive X-ray diffraction; FTIR, Fourier transform infrared spectroscopy.
